# Using Taxol-sensitized budding yeast to investigate the effect of microtubule stabilization on anaphase onset

**DOI:** 10.1016/j.xpro.2023.102522

**Published:** 2023-08-18

**Authors:** Angela R. Bunning, Samuel J. Anderson, Mohan L. Gupta

**Affiliations:** 1 Department of Genetics, Development, and Cell Biology, Iowa State University, Ames, IA 50010, USA

**Keywords:** Cell Biology, Cell-based Assays, Genetics, Model Organisms

## Abstract

The microtubule (MT)-stabilizing drug Taxol (paclitaxel) is a commonly used tool to investigate MT dynamics and MT-dependent processes. Here, we present a protocol for using Taxol-sensitized budding yeast to investigate the effect of microtubule stabilization on anaphase onset. We describe steps for establishing a log phase culture, synchronizing cells in G1, arresting in metaphase, and releasing cells into Taxol. We then detail procedures for imaging and scoring anaphase onset. This protocol facilitates maintenance and reproducibility in testing drug-sensitized and Taxol-sensitized yeast strains.

For complete details on the use and execution of this protocol, please refer to Proudfoot et al.^1^

## Highlights

• Taxol-sensitized budding yeast allows rapid stabilization of spindle microtubules
• Monitor effect of unattached and/or low-tension kinetochores on anaphase onset timing
• Allows reduced tension at attached kinetochores in fully formed spindles
• Determine the requirement of specific genes in tension-specific kinetochore signaling

## Abstract (editor highlights)

Publisher’s note: Undertaking any experimental protocol requires adherence to local institutional guidelines for laboratory safety and ethics.

## Graphical abstract

**Figure:**
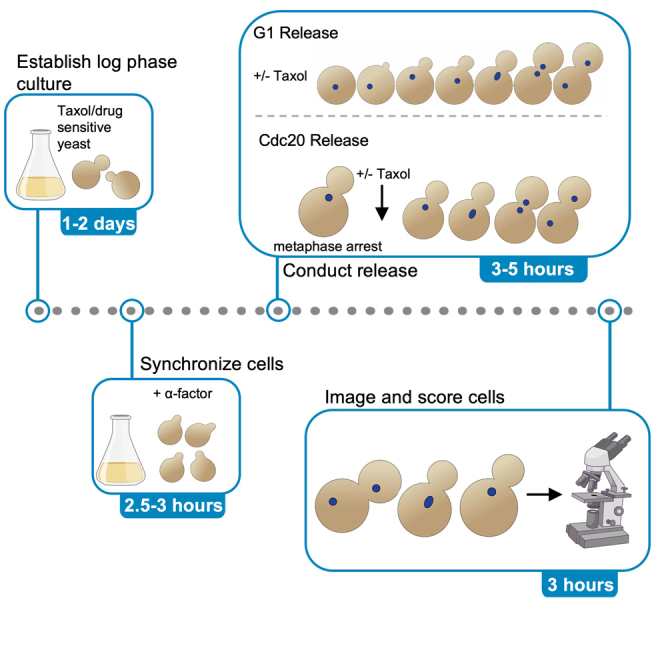

## Before you begin

During mitosis, a vital MT-dependent process is chromosome segregation, when MTs that make up the mitotic spindle pull sister chromatids apart equally into the two daughter cells. MTs attach to chromosomes at kinetochores, where they exert pulling forces known as tension on the attached chromosomes via MT depolymerization. While kinetochore-MT attachments are essential for accurate chromosome segregation, the mechanism of how tension promotes accurate chromosome segregation is unclear. Budding yeast cells treated with MT-stabilizing drugs decrease MT-generated pulling forces, creating an experimental system to investigate how kinetochores with low tension impact anaphase onset timing. Wild type budding yeast are not sensitive to Taxol,^2^^,^^3^ therefore budding yeast strains used in these assays are sensitized to small molecules in general by gene deletions (*pdr1Δ, pdr3Δ, erg6Δ*) and to Taxol specifically by point mutations in β-tubulin (*tub2*-A19K-T23V-G26D-Y270F).^1^^,^^4^ Deletions of the transcription factors that regulate the pleotropic drug pump response (*pdr1Δ* and *pdr3Δ*) and a gene that normally reduces cell permeability (*erg6Δ*) decrease the yeast cell’s ability to reduce intracellular Taxol levels,^5^ while the β-tubulin point mutations create an active Taxol binding pocket not found on wild type yeast tubulin.^6^ Other drug-sensitized strains may harbor different mutations that analogously make them susceptible to variations in experimental conditions that normally do not affect non-sensitized yeast strains. For the strains used in the methods described below, drug-sensitized cells refers to strains that have only the pleotropic drug response and cell permeability mutations (*pdr1Δ*, *pdr3Δ*, and *erg6Δ*) but lack the β-tubulin point mutations. Taxol-sensitized cells refers to strains that have the pleotropic drug response and cell permeability mutations (*pdr1Δ*, *pdr3Δ*, *erg6Δ*) and the β-tubulin point mutations. The methods provided for two anaphase onset timing assays and a Taxol sensitivity plating assay will help to limit the variations in experiments that can occur using Taxol-sensitized cells. These methods are important in performing assays that will help to understand how certain genes implicated in chromosome segregation can regulate anaphase onset timing in cells with decreased tension at kinetochores.

See the limitations section of this paper for detailed information about conditions affecting cultures, plates, and imaging conditions with Taxol.

### Preparing optimized Taxol stock for yeast cultures

**Timing: 30 min**

The following steps will help to make a reliable 1 mM Taxol stock that will be used in the following assays. We provide steps to ensure accurate steps to measure Taxol concentration in the stock solution, as well as tips for stock solution storage.

1. Measure ∼8 mg Taxol powder into a 1.5 mL microfuge tube.

***Note:*** If you purchase a 5 or 10 mg bottle of Taxol it may be best to resuspend the entire amount in the original bottle, using the corresponding volume of DMSO and then determine the concentration as described below.

2. Dissolve Taxol power in DMSO (∼3 mL).

***Note:*** Be sure to dissolve all the powder in the tube by adding 1 mL DMSO at a time, mixing, then transferring each 1 mL into a 14 mL tube.

3. Dilute Taxol/DMSO solution in methanol (1:20) based on the volume appropriate for the size of cuvette used to measure absorbance.
  a. e.g., our cuvettes are 1 mL total, 950 μL methanol, 50 μL Taxol/DMSO solution.
4. Dilute DMSO in methanol (1:20) as a blank (1 mL total blank solution, 950 μL methanol, 50 μL DMSO).
5. Measure the absorbance of the Taxol solution relative to the blank at 273 nm.

***Note:*** Methanol should be used as a blank for dilution of DMSO/Taxol solution because methanol does not absorb at 270 nm, unlike DMSO. Taxol is also highly soluble in methanol and will prevent precipitation while measuring absorption.

**CRITICAL:** Ensure the cuvettes are UV transmissible.

6. Calculate the stock concentration using the extinction coefficient of 1700 M^−1^cm^−1^.^7^

$$\left[Taxol\right]=\left(A_{273}\times 20/1700\;M^{-1}{cm}^{-1}\right)\times 1000\;mM/M\;Taxol$$

The output of this concentration will be C_1_ in the relationship C_1_ × 2.95 mL = 1 mM × V_final_

7. Dilute the Taxol/DMSO solution to 1 mM stock with additional DMSO to reach V_final_.

**CRITICAL:** If you do not have 3 mL dissolved Taxol/DMSO solution adjust the relationship with C_1_ accordingly.

8. Ensure the stock solution is stored in an airtight container at least −20°C.
9. Remake a fresh stock solution if any small portion of the stock remains unfrozen, e.g., liquid droplet at −20°C.

***Note:*** It stores best in 2 mL cryo-tubes with sealing caps, but it can also be stored in a screwcap 14 mL tube with a parafilm seal if a larger volume is needed.

### Making methyl cellulose solution for Taxol cultures

**Timing: 1–4 h**

Taxol is poorly soluble in aqueous solution. The efficacy of Taxol is increased on agar plates, likely due to the agar promoting solubility and increasing the effective concentration of Taxol. Supporting this idea, methyl cellulose shares structural similarities with agar, remains liquid at culture temperatures, and similarly is required to increase the efficacy of Taxol in liquid media.

10. Weigh out enough methyl cellulose (viscosity: 4,000 cP) to make a 2% w/v solution of desired volume.
11. Place room temperature (around 24°C) or cold Milli-Q water and a clean stir bar in autoclavable media bottle.
12. Begin stirring.
13. Add methyl cellulose slowly to the Milli-Q water.
14. Stir until completely dissolved and the solution is translucent.
15. Autoclave for 20 min once dissolved.
16. Remove from autoclave and let cool before proceeding to the next step.

***Note:*** After autoclaving, methyl cellulose will appear solid while hot and will become liquid again when cooled.

17. Aliquot into 1.5 mL microfuge tubes or microfuge tubes with o-ring caps.
18. Store at −20°C.
  a. Can be stored long term at −20°C (9 months–1 year).

**CRITICAL:** The methyl cellulose stock must be stored frozen.

### Making the 2 mg/mL methionine solution for Cdc20 arrest

**Timing: 40 min**

Methionine is used to control the levels of *CDC20* during the Cdc20 arrest assays, and the following steps will instruct you how to make an appropriate stock solution.

19. Weigh out 200 mg L-methionine
20. Add 75 mL of Milli-Q water to bottle with a stir bar.
21. Add methionine to water.
22. Stir until dissolved.
23. Bring up solution to 100 mL.
24. Autoclave for 20 min.
25. Store short term at room temperature (around 24°C )(< 2 months). For long term storage (> 2 months), move to 4°C.

## Key resources table

**Table:** 

REAGENT or RESOURCE	SOURCE	IDENTIFIER
**Chemicals, peptides, and recombinant proteins**		

Methyl cellulose (viscosity: 4,000 cP)	Sigma Life Sciences	Product #M0512
Taxol (paclitaxel, >99.5%)	TSZ CHEM	Cat# ROS036
L-Methionine, 98+%	Acro Organics	Code: 166161000
Yeast nitrogen base without amino acids	BD Difco™	Ref# 291940
Alpha-factor mating pheromone (10 mM in 0.1 M sodium acetate pH 5.2)	Zymo Research	Cat# Y1001
Bacto peptone	BD Bacto™ Peptone	Ref# 211705
Synthetic Complete Media	Sunrise Science Products	Cat# 1300-030
SC-MET Media	Sunrise Science Products	Cat # 1343-030
Propidium iodide (0.5 mg/mL solution)	Roche	11819000

**Experimental models: Organisms/strains**		

*S. cerevisiae*: Strain background S288C; See Table S1	N/A	S288C

## Step-by-step method details

### G1 release assay

**Timing: 3 days**

The G1 release assay is used to determine the effect of tensionless and unattached kinetochores on the timing of anaphase onset. In Taxol-sensitive cells, Taxol suppresses MT dynamics during spindle formation leading to delays in establishing MT-kinetochore attachments. The populations of kinetochores within the cells that are unattached and tensionless, or attached with low tension cause a delay in anaphase onset.

#### Establishing a log phase culture

1. Inoculate 5 mL SC media (Synthetic Complete) with Taxol-sensitive yeast strain (MGY 2150; Table S1).
  a. Grow this culture at 30°C for 12–24 h before using as a pre-culture to inoculate larger cultures.
2. Use the freshly saturated 5 mL culture to inoculate into a 30–50 mL culture, also in SC media at 30°C, at a dilution that will be in log phase with an OD_600_(0.15–0.4) the next morning.
3. Pellet the cells by centrifugation (3,000 g for 3–5 min) once desired OD is reached.

***Note:*** If the culture is undergrown at this point, let it continue growing until it reaches an OD_600_(0.15–0.4). If culture is overgrown, it's best to prepare another 5 mL starter culture and try again. A possibility is to start multiple 30–50 mL cultures with different dilutions of inoculum to ensure one is in the proper stage in the morning.

***Note:*** If you plan to fix cells for scoring anaphase onset via DAPI staining, put a bottle of 70% ethanol in the freezer the night before the assay to ensure it is ice-cold for fixing samples. See Table 1 to plan accordingly based on the downstream assay.

**Table 1:** Downstream applications and processing of cells from G1 and Cdc20 Release assays

Application	Processing steps
Determine anaphase onset via scoring DAPI separation	1. Spin down ethanol-fixed cells in tabletop centrifuge, decant, and wash with 1 mL 1× PBS (solution made according to Cold Springs Harbor Protocols). Repeat this PBS wash once. 2. Suspend pellet in 300–500 μL low concentration DAPI solution (≤50 ng/mL) in 1× PBS. 3. Cells can be stored before imaging at 4°C for several weeks and for longer at −20°C.
Determine spindle morphology via fluorescent marker	1. Spin down cells in tabletop centrifuge, decant, and fix with 500 μL of 3.7% formaldehyde. Place tubes in dark, room temperature place (e.g., a drawer). Alternatively, for more gentle treatment of spindles, 37% formaldehyde stock can be diluted directly into each sample tube to a final volume of 3.7%. 2. Fix cells for ∼15 min, no longer than 20 min. If cells are fixed longer, fluorescence signal will deteriorate. 3. Spin down and wash two times with 1 mL PBS. 4. Spin down and suspend cells in ∼300–500 μL of PBS. 5. Cells can be stored before imaging at 4°C for several days and for longer at −20°C.
Determine DNA content via FACS	1. Spin down ethanol-fixed cells in tabletop centrifuge, decant, and resuspend cell pellet in 1 mL of 0.20 mg/mL of RNAase A. 2. Leave at 37°C overnight (can leave longer if need be). 3. Remove from 37°C, spin down cells and decant. 4. Suspend in 1 mL of 5 μg/mL propidium iodide (1:100 dilution of 0.5 mg/mL in PBS. 5. Incubate samples in propidium iodide solution for at least 3 h in dark at 4°C. 6. Use directly for FACS analysis. Propidium iodide (PI) intercalates into DNA, thus the PI intensity correlates with DNA content. Also please note that PI staining is only applicable in cells with damaged membranes, which results from the ethanol-fixation.

#### Synchronizing culture in G1

4. Resuspend the cell pellet in 10 mL SC media.
5. Add 3.5 μL alpha-factor (3.5 μM final concentration alpha-factor) to arrest the culture in G1.

***Note:*** This resuspension can be done in a culture tube or a small flask.

6. Incubate and shake/rotate (230–250 rpm) these cells at 30°C for 3 h.
  a. Check under a light microscope to be sure they have been arrested (i.e., >95% unbudded and/or schmooing).
  b. The cells should start to take on an oval/teardrop shape. If there are still small-budded cells seen on the slide, let the culture arrest for another 15–30 min and recheck.
  c. Label 10 microfuge tubes per condition for each time point: 0, 60, 90, 120, 150, 165, 180, 195, 210, and 240 min.

***Note:*** For +/- Taxol, at each designated time point, there should be two sets of tubes, one for the Taxol-treated and one for the control strain.

7. Take ∼100 μL of the culture, spin down in 1.5 mL microfuge tube, and fix with 500 μL ice-cold 70% ethanol.
8. Label this tube time point 0.

***Note:*** It is possible that some genetic modifications increase genetic instability and/or cell death. These cultures may contain a percentage of dead/dying cells that appear not to arrest in G1. For genotypes that have genetic instability, this can be verified using analogous non-sensitized strains and/or staining for live/dead cells.

#### Releasing culture from G1 into Taxol

9. Spin down the alpha-factor arrested culture.
  a. Transfer the pellet to a microfuge tube.
  b. Wash 3 times with 1 mL Milli-Q water.
  c. Decant the supernatant from the second wash.
  d. Resuspend the cell pellet in 1 mL.
  e. Split the cell suspension equally (500 μL:500 μL) between two tubes.
10. Spin down the cells for a final time and decant supernatant.

**CRITICAL:** Wash the cells with Milli-Q water as quickly as possible to ensure a timely and synchronous release from the alpha-factor arrest. Troubleshooting 1

11. Resuspend each pellet and bring to a total of 5 mL in SC media containing 0.02% methyl cellulose (50 μL, or 1:100 of 2% stock) with and without 30 μM Taxol (150 μL of 1 mM stock, or equivalent amount of DMSO in control).
12. Conduct the release at around 24°C by keeping the cultures on the bench.
  a. Swirl the cultures by hand every ∼10 min for the entire assay to ensure the cells remain suspended.
13. Take 500 μL of each culture at the designated time points and place in the appropriately labeled tube prepared in Step #6c above.
14. Quickly spin down the cells (4,000 g for 30 s).
  a. Rapidly fix them according to the downstream application of the cells (see Table 1).
15. Add 1.5 μL alpha-factor (3.8 μM final alpha-factor concentration) after the 90-min time point to each release tube, control and Taxol-treated, to prevent post-anaphase cells from re-budding.

#### Imaging and scoring anaphase onset

**Figure 3:**
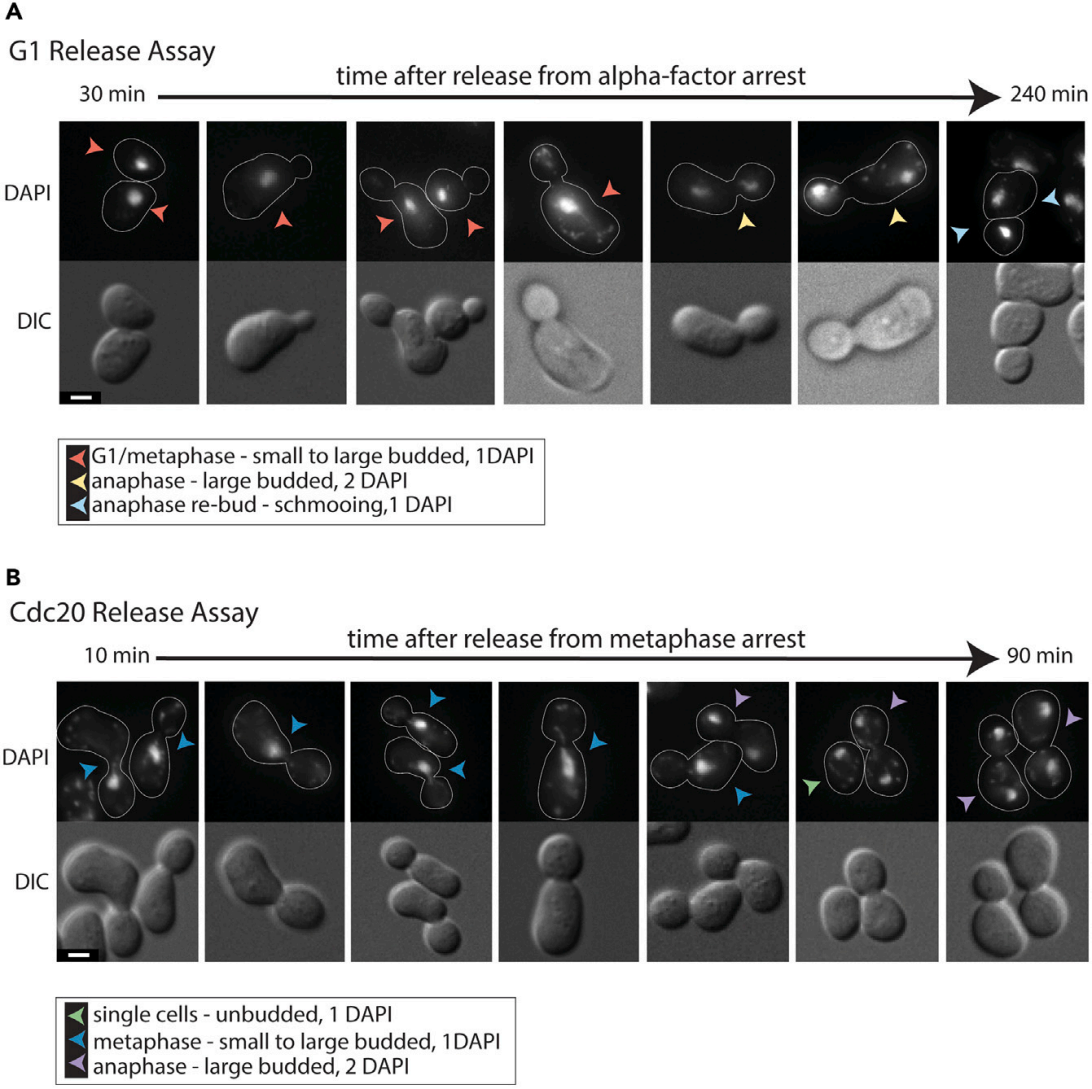
Examples of DAPI-stained cells from both release assays (A and B) Representative images of DAPI-stained cells from (A) G1 (MGY2150) and (B) Cdc20 (MGY1293) release assays. Cells were imaged with 12 z-planes at 0.5 μm intervals using DIC and DAPI settings, and maximum z-projection images are shown for DAPI, and a single focal plane for DIC. Cells scored in the G1 release assay (A) are grouped into three categories based on cell and DNA morphology: G1/metaphase - small to large budded with 1 DAPI focus, anaphase - large budded with 2 DAPI foci, and anaphase re-bud / schmooing cells with 1 DAPI focus (seen in later time points). In the Cdc20 release assay (B), cells are grouped into three similar categories: single cells - unbudded with 1 DAPI focus, metaphase - small to large budded with 1 DAPI focus, and anaphase - large budded with 2 DAPI foci. If a large budded cell has a DAPI signal that is dumbbell shaped but still connected, it is counted as a metaphase cell in both the G1 and Cdc20 release assays. Scale bars = 2 μm.

16. Stain cells with DAPI (see Table 1).
17. Score anaphase onset by the state of DAPI separation.
  a. The categories are pre-anaphase (1 DAPI focus, small or large budded), anaphase (2 DAPI foci, large budded), or anaphase re-bud (1 DAPI focus, schmooing) (Figure 3).

***Note:*** DAPI can stain mitochondrial DNA under certain conditions, which is why low concentrations of DAPI should be used to ensure nucleus staining is the dominant signal while imaging.

18. Take images with 12 planes (∼0.5 μm separation) in the DAPI channel (excited at 387, emission at 447) and Differential Interference Contrast (DIC) to ensure that each DAPI focus can be scored as separated or connected.
  a. If DIC is not available on your microscope, polarized or bright field transmitted light is also suitable for imaging. See Figure 3A for examples of each cell category and how they would be scored.
19. Score 150–200 cells for each time point.

### Cdc20 release assay

**Timing: 3 days**

The Cdc20 release assay is used to measure the effect of attached kinetochores with reduced tension on the timing of anaphase onset. To generate a metaphase spindle with kinetochores that are attached to MTs and under tension, the cells are arrested in metaphase via depletion of *CDC20*, in the absence of MT poisons. Due to the fact *CDC20* is an activator of the Anaphase Promoting Complex (APC/C), when it is depleted, cells cannot enter anaphase.^8^ The strain used in this assay (MGY1293) has endogenous *CDC20* under the control of a methionine-inducible promoter.^9^ When methionine is present in the media, Cdc20 levels will be depleted, and the cells arrest in metaphase. Taxol is added once the culture is arrested in metaphase (pMET3-Cdc20 depletion) where the spindles are fully formed, and kinetochores have established bipolar MT attachments. Taxol treatment suppresses MT dynamics and decreases the resultant tension forces generated at MT-kinetochore attachment sites.

#### Establishing log phase culture

20. Inoculate a 5 mL SC-MET media (SC media lacking methionine) culture (MGY 1293; Table S1).
  a. Grow this culture at 30°C for 12–24 h before using to inoculate larger overnight cultures.
21. Use the freshly saturated 5 mL culture to inoculate into a 30–50 mL culture, also in SC-MET media at 30°C, at a dilution that will be in log phase with an OD_600_(0.15–0.4) the next morning.
22. Pellet the cells by centrifugation (3,000 g for 3–5 min) once desired OD is reached.

***Note:*** If the culture is undergrown at this point, let it continue growing until it reaches an OD_600_(0.15–0.4). If culture is overgrown, it's best to prepare another 5 mL starter culture and try again. A possibility is to start multiple 30–50 mL cultures with different dilutions of inoculum to ensure one is in the proper stage in the morning.

#### *Synchronizing culture in G1*

23. Collect the overnight culture by centrifugation.
24. Resuspend pellet in 10 mL SC-MET media.
25. Add 3.5 μL alpha-factor (3.5 μM final concentration alpha-factor) to arrest the cells in G1.

***Note:*** This resuspension can be done in a culture tube or a small flask.

26. Incubate and shake/rotate (230–250 rpm) these cells at 30°C for 3 h.
  a. Check under a light microscope to be sure they have arrested (i.e., >95% unbudded and/or schmooing).
  b. The cells should start to take on an oval/teardrop shape. If there are still small-budded cells seen on the slide, let the culture arrest for another 15–30 min and recheck.
27. Set up metaphase arrest cultures while cells are arresting in G1.
  a. Metaphase-**arrest** cultures: Make two identical tubes of metaphase-arrest media, 970 μL each in glass culture tubes for each genotype.
    i. Each tube contains: 860 μL SC-MET, 10 μL 2% methyl cellulose (1:100 dilution), 100 μL 2 mg/mL methionine solution (1:10 dilution). See troubleshooting 2 and troubleshooting 3 for more details about the effect of methionine concentration on release.
  b. Metaphase-**release** cultures: Make two tubes of metaphase-release media, 5 mL each in glass culture tubes for each genotype.
    i. One control tube: 4.8 mL SC-MET, 50 μL 2% methyl cellulose (1:100 dilution), 150 μL DMSO.
    ii. One Taxol tube: 4.8 mL SC-MET, 50 μL 2% methyl cellulose (1:100 dilution), 150 μL 1 mM Taxol.
  c. Label two sets of 9 microfuge tubes, one for control and one for Taxol treated cells (10, 20, 30, 40, 50, 60, 70, 80, 90 min) for each genotype.

***Note:*** If you plan to fix cells for scoring anaphase onset via DAPI staining, put a bottle of 70% ethanol in the freezer the night before the assay to ensure it is ice-cold for fixing samples. See Table 1 to plan accordingly based on the downstream assay.

***Note:*** For +/- Taxol, at each designated time point, there should be two sets of tubes, one for the Taxol-treated and one for the control strain.

#### Releasing from G1 and arresting in metaphase

28. Spin down the alpha-factor arrested culture.
  a. Transfer the pellet to a microfuge tube.
  b. Wash 3 times with 1 mL Milli-Q water.
  c. Decant the supernatant from the second wash.
  d. Resuspend the cell pellet in 1 mL.
  e. Split the cell suspension equally (500 μL:500 μL) between two tubes.
29. Spin down the cells for a final time.
30. Decant supernatant.

**CRITICAL:** Wash the cells with Milli-Q water as quickly as possible to ensure a timely and synchronous release from the alpha-factor arrest. (Troubleshooting, problem 1)

31. Resuspend each of these pellets into the 970 μL metaphase-**arrest** media (step 27a above) and incubate for 1.25–1.5 h on a rotator at 30°C to reach metaphase arrest.
  a. Check the cells on a light microscope to confirm they bud synchronously and reach medium-budded state.
  b. If not, let the cells incubate longer.
  c. If the cells still don’t seem arrested, stop assay here and check out troubleshooting 3.
32. Add 30 μM Taxol final concentration (30 μL of 1 mM stock) to one tube, and 30 μL DMSO to the control tube once the cells appear medium to large budded.
33. Incubate on the bench for another 15 min (gently shaking/swirling by hand every few minutes).

#### Releasing from metaphase

34. Spin down cultures in a microfuge tube.
35. Quickly wash cells 3 times with Milli-Q.

**CRITICAL:** Wash the cells with Milli-Q water as quickly as possible to minimize the potentiality that cells expel Taxol and MTs transiently generate tension.

36. Resuspend one of each genotype pellet in the 5 mL metaphase-**release** media containing Taxol, and the other pellet in the control metaphase-**release** media.
37. Keep the two 5 mL release cultures on the bench around 24°C.
  a. Gently shake/swirl the cultures by hand every ∼10 min throughout the entire assay to ensure the cells remain suspended.
38. Take 500 μL of each culture and place in the appropriately labeled tube at the designated time points.
39. Quickly spin down the cells (4,000 g for 30 s).
  a. Rapidly fix according to the downstream application of cells (see Table 1).

#### Imaging and scoring anaphase onset

40. Stain cells with DAPI (see Table 1).
41. Score anaphase onset by the state of DAPI separation.
  a. The categories are unbudded cells (1 DAPI focus, unbudded), metaphase (1 DAPI focus, medium to large budded), and anaphase (2 DAPI foci, large budded).
42. Take images with 12 planes (∼0.5 μm separation) in the DAPI (excited at 387, emission at 447) and DIC channels to ensure that each DAPI spot can be scored as separated or connected.
  a. If DIC is not available on your microscope, polarized or bright field transmitted light is also suitable for imaging. See Figure 3B for examples of each cell category and how they would be scored.
43. Score ∼150–200 cells for each time point.

### Taxol sensitivity spotting assay

**Timing: 5 days**

A Taxol sensitivity spotting assay is used to investigate if your genes of interest are needed for sensing tension, and if they can delay anaphase onset in the presence of low tension kinetochores. For cells that are plated on Taxol-containing media, if they have gene deletions that cause impaired kinetochore tension sensing, they will be more sensitive to Taxol than Taxol-sensitized control cells.

#### Making Taxol-containing plates

**CRITICAL:** The volume and relative duration of autoclaving and cooling of yeast YPD media alters the effectiveness of Taxol, likely by affecting solubility. To standardize this, we filter-sterilize YPD media to be used with Taxol.

**Table:** 2× YPD media for final volume of 600 mL

Reagent	Final concentration	Amount
Bacto Peptone	2×	12 g
Yeast extract	2×	6 g
Dextrose	2×	12 g
Milli-Q	N/A	up to 300 mL
**Total**	**2**×	**300 mL**

44. Calculate the amount of YPD media needed for all plates (35 mL for each plate, plus a small excess).
45. Dissolve the corresponding amount of YPD media ingredients in half the final volume of Milli-Q water (2× YPD).
  a. The components and amounts needed for 2× YPD are listed in the table. These amounts would be used to generate a final volume of 600 mL of YPD plus agar for plates.
46. Add the corresponding amount of agar (for a final volume of 600 mL, 15 g of agar would be added to 300 mL of Milli-Q) and a stir bar to the same volume of Milli-Q water (2× agar) to a bottle.
47. Autoclave.
48. Filter-sterilize the 2× YPD solution into the molten 2× agar using a 0.22 μm bottle-top filter.

**CRITICAL:** The vacuum used to filter-sterilize will significantly cool the 2× YPD liquid and subsequently the 2× agar solution. Therefore, do not allow the 2× agar solution to cool much before this process. Otherwise, the combined mixture will solidify before plates can be poured.

***Note:*** No issues have been observed with Taxol being added to hot media.

49. Thoroughly mix the 2× YPD and 2× agar to create 1× final using the stir bar and stir plate.
50. Pour 35 mL into a 50 mL conical tube and add the corresponding amount of Taxol stock solution and DMSO before cooling.
51. See Table 2 for an example of appropriate amounts of Taxol stock solution and DMSO to be added for the desired range of Taxol concentrations.
  a. Because the 1 mM Taxol stock is prepared in DMSO, the total amount of DMSO added for the highest concentration plate needs to be added to all the assay plates, regardless of their individual Taxol concentrations.
    i. Example: To pour a plate with a final Taxol concentration of 2.5 μM, while the highest assay concentration plate to be poured is 10 μM.
    ii. Starting with 350 μL (the highest amount of Taxol stock used in the 10 μM plate) – 88 μL Taxol stock in DMSO (amount of Taxol stock used in 2.5 μM plate) = 262 μL of additional DMSO must be added to the 2.5 μM plate.

**Table 2:** Example of a Taxol mixing table for 35 mL plates

Final Taxol concentration in plate (μM)	Amount of 1 mM Taxol stock (μL)	Amount of supplemental DMSO (μL)
0	0	350
0.5	18	332
1.0	35	315
1.5	53	297
2.0	70	280
2.5	88	262
3.0	105	245
3.5	123	227
4.0	140	210
4.5	158	192
5.0	175	175
5.5	193	157
6.0	210	140
7.0	245	105
8.0	280	70
9.0	315	35
10.0	350	0
52. Close the tube and invert gently 5 times to mix then pour into a petri dish for each plate concentration.
53. Allow plates to dry rapidly with lid off for ∼30 min in a laminar flow hood and use within one day if possible.
  a. If a hood is not possible, realize that plates should be used within 1–2 days and be of identical age.

**CRITICAL:** See the information about pouring Taxol plates in the Limitations section. There is critical information about the relative effectiveness of Taxol in plates, notes about preparing the YPD for the Taxol plates, peptone brand and lots, and the age of the Taxol plates, which can all be sources of variation in this assay.

#### Establishing a saturated culture for spotting

54. Inoculate a 5 mL culture (MGY1872, MGY1874, and MGY2104; Table S1) in YPD.
  a. Incubate on a rotator/shaker overnight at 30°C.
55. Use this culture to inoculate 10 μL into 10 mL fresh YPD the next day.
  a. Incubate on a rotator/shaker at 30°C for the next 48–72 h.

***Note:*** We chose to use saturated cultures for spotting assays because some mutant cultures grow slower than control cultures. To generate high density starting spots these cultures need additional growth time and using 48–72 h cultures results in similar culture densities.

56. Spot fully grown cultures onto the Taxol-containing plates.
  a. If the control plate is not showing similar density between strains, then standardize cultures to the same OD_600_ before serial dilutions.

#### Spotting the Taxol plates

57. Add 150–200 μL of each saturated culture to the top well in a 96-well plate.
58. Perform 10× serial dilutions to create the range of dilutions for plating.
  a. To create each spot a multichannel pipette is used with 2–3 μL of each 10× serial dilution.
  b. Alternatively, a multi-pinned spotter, or ‘frogger’ can be used to transfer droplets from the 96-well plate onto the various test plates.
59. Incubate spotted plates at 24°C and monitor for differences in Taxol sensitivity over the next 3–5 days.
  a. Be sure that any plates between different spotting assays that are being compared have the same amount of growth time at identical temperatures.

***Note:*** It is possible that intermediate concentrations of Taxol could result in levels of cell death that are obvious in early stages of colony growth but become obscure in later days when control colony growth slows and the inhibited strain colonies ‘catch up’ in size.

***Note:*** The spotting assay can be performed at different temperatures, e.g., 30°C. However, we found that on control plates, without drug, colony formation of bub1 mutant strains lags that of strains harboring functional Bub1. This difference is less pronounced at 24°C, presumably due to increased time to complete spindle assembly.

60. Image the plates with an overhead bright light.
61. Record and analyze results.

## Expected outcomes

In both release assays, when assessing the effect of Taxol on cell cycle timing, DMSO-treated (control) cells should synchronously enter anaphase while Taxol-treated cells have delayed anaphase entry

**Figure 1:**
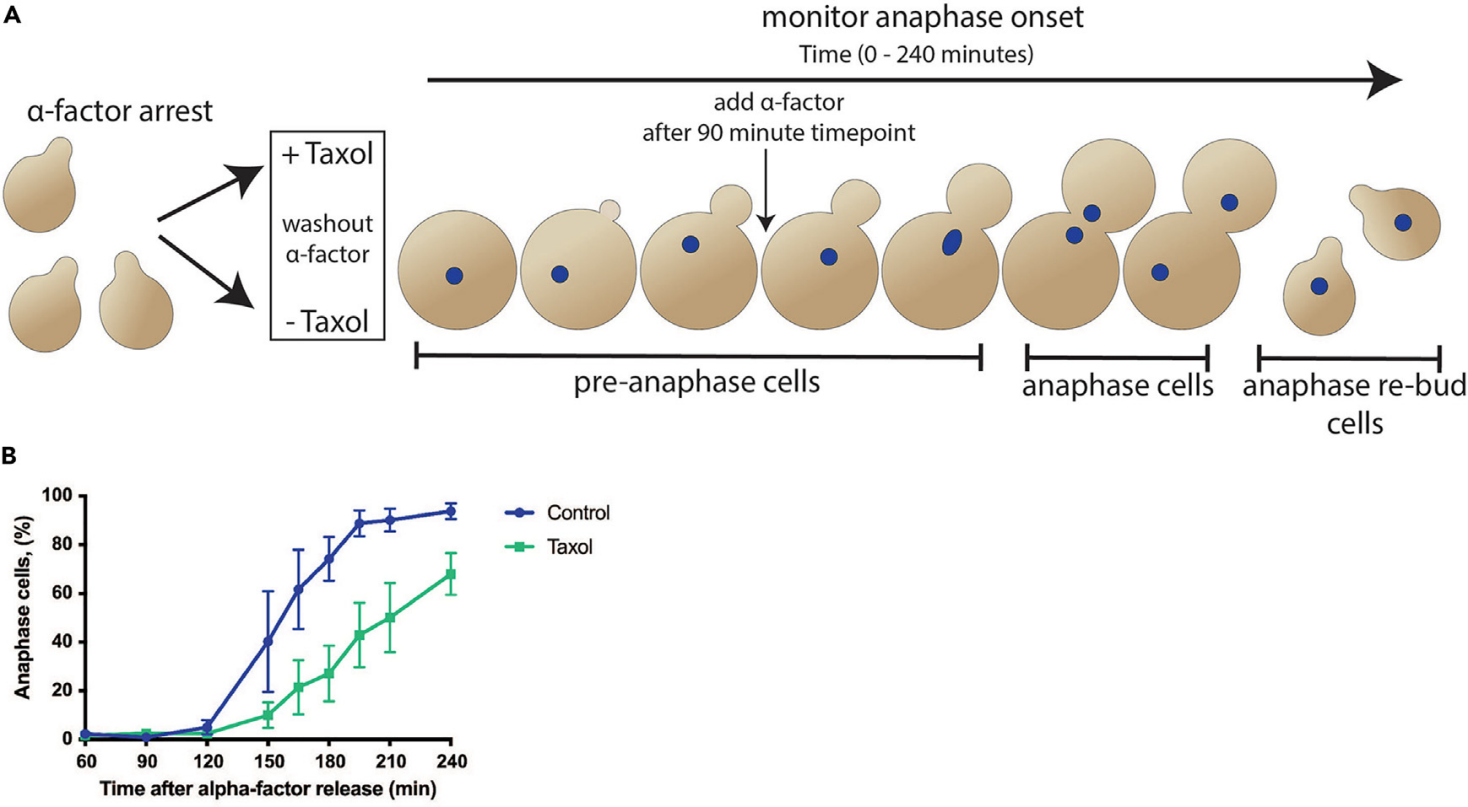
G1 release assay (A) Schematic of G1 release assay (MGY2150). The G1 release assay monitors the timing of anaphase onset when tensionless and unattached kinetochores are induced by Taxol treatment during spindle assembly. Cells are first synchronized in G1 using alpha-factor before being split into two cultures. Upon release from alpha-factor arrest one is treated with Taxol in DMSO and the other with only DMSO as a control. Samples are withdrawn and fixed over the subsequent 240 min (0, 60, 90, 120, 150, 165, 180, 195, 210, 240 min) and used to monitor anaphase onset via DAPI staining of DNA (blue spots). Note that if alpha-factor is included at 90 min post release from G1 the anaphase re-bud cells will be schmooing. If alpha-factor is omitted they will be small-budded. (B) Results from the G1 release assay where symbols represent the mean ± SEM from 3 different experiments. Percent anaphase cells at each time point is calculated by (number of anaphase large budded cells + anaphase re-bud cells) / the total number of cells scored in the time point ∗ 100. For each timepoint 100–200 cells were scored per experiment.

**Figure 2:**
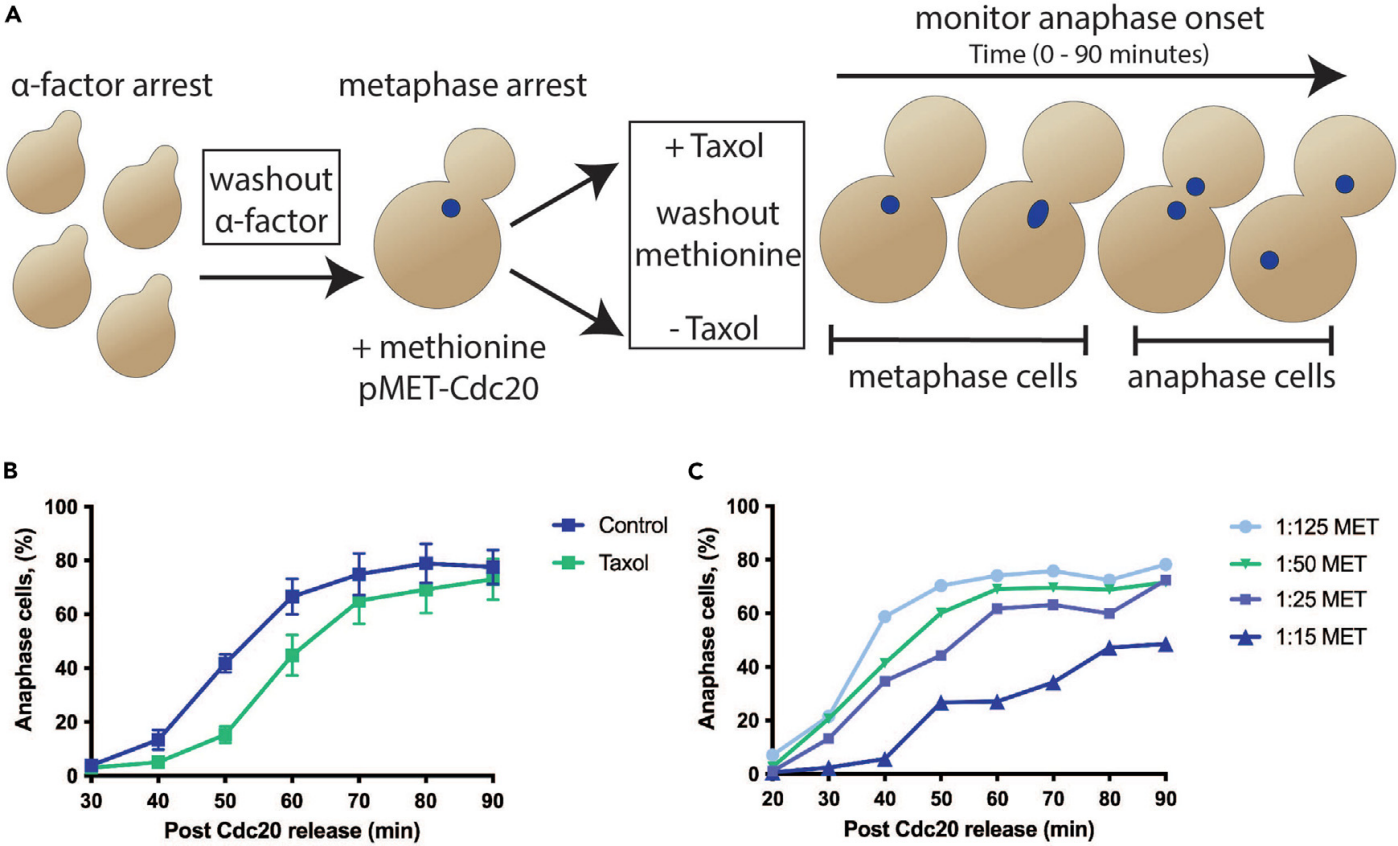
Cdc20 release assay (A) Schematic of Cdc20 release assay (MGY1293). The Cdc20 release assay monitors the effect of attached kinetochores with reduced tension on the timing of anaphase onset by treating cells with Taxol only after spindle assembly is complete. This isolates the effect of low tension on attached kinetochores. Cells are first synchronized in G1 using alpha-factor, and then released and subsequently arrested in metaphase by Cdc20 depletion. This metaphase arrest allows for complete spindle formation and establishment of bipolar attachments with unperturbed microtubule dynamics before Taxol treatment. Cells are then released into media containing Taxol in DMSO, or only DMSO as a control, and samples are withdrawn and fixed over the next 90 min to monitor anaphase onset via DAPI staining of DNA (blue spots). (B) Results from the Cdc20 release assay where symbols represent the mean ± SEM from 3 different experiments. Percent anaphase cells at each time point is calculated by the total number of anaphase large budded cells / (anaphase large budded cells + metaphase cells) ∗ 100. For each timepoint 100–200 cells were scored per experiment. (C) If scaling up the volumes in this assay, the amount of methionine used in the metaphase arrest can affect the speed and synchronicity of metaphase release into anaphase. Keep the ratio of total methionine (i.e., concentration ∗ volume) to cell number similar to the original Cdc20 release volumes and adjust accordingly if needed.

When a mutation of interest is introduced to a Taxol-sensitized strain, and the same assays are performed, the effect on anaphase onset can be assessed by comparing the results with the mutant strain to those from the DMSO vs. Taxol of the control strain. For example, deletion of certain Spindle Assembly Checkpoint (SAC) genes such as *BUB1* or *MAD1* can alter the timing of anaphase onset compared to control Taxol-sensitized cells.^1^ Figure 3 illustrates examples of DAPI-stained, control Taxol-sensitized cells that are scored to determine anaphase onset (Figures 1B and 2B).

**Figure 4:**
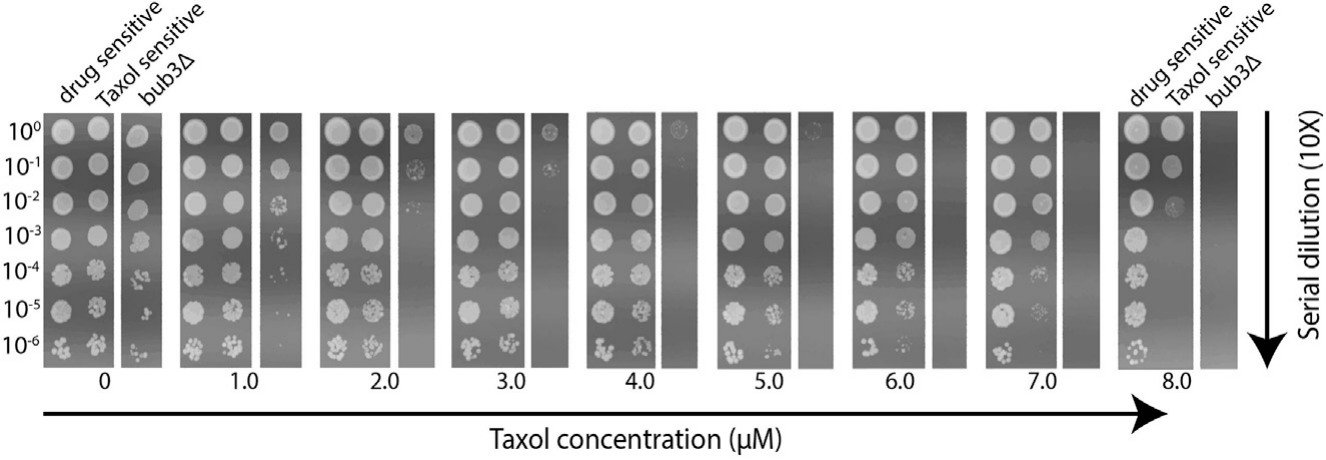
Taxol sensitivity spotting assay Example of a Taxol sensitivity spotting assay with Taxol concentration increasing from left to right and culture density decreasing 10-fold with each descending spot. Plates were made fresh as described and allowed to dry under a hood before being spotted with 2.2 μL of each dilution from 2-day saturated cultures. These plates were spotted with drug sensitive cells (MGY1872, *pdr1Δ pdr3Δ erg6Δ*) containing wildtype tubulin, Taxol sensitive cells (MGY1874*, tub2-A19K-T23V-G26D-Y270F pdr1Δ pdr3Δ erg6Δ*) containing Taxol-sensitized tubulin, and Taxol sensitive cells lacking the spindle checkpoint protein, Bub3 (MGY2104, *tub2-A19K-T23V-G26D-Y270F, pdr1Δ pdr3Δ erg6Δ bub3Δ*).

The Taxol sensitivity spotting assay will demonstrate if your mutation(s) of interest is involved in delaying anaphase onset due to the effects of Taxol. When cells are challenged with higher concentrations of Taxol, MTs become increasingly stabilized, and more time is required to build the spindle and for kinetochores to establish bioriented attachments to MTs. Suppose Taxol-sensitized cells that include your mutation(s) of interest prove more sensitive to Taxol than the Taxol-sensitized bub3Δ cells (Figure 4). In that case, it could be that your gene(s) of interest is needed to delay anaphase onset to allow sufficient time to complete spindle assembly.

## Limitations

### Using Taxol in liquid media cultures

Sensitivity in yeast cultures requires Taxol concentrations exceeding its solubility in aqueous solutions, up to 30 μM. For Taxol to be effective at these concentrations, it requires addition of methyl cellulose to liquid media. This is most apparent in SC media. For liquid cultures, 0.02% final methyl cellulose is added (1:100 dilution of 2% stock) **prior to** Taxol addition. Without use of methyl cellulose, or adding Taxol before methyl cellulose, there is a precipitous decrease in the effectiveness of Taxol (data not shown). See troubleshooting 4 for more details.

### Preparing consistent plates for Taxol sensitivity spotting assays

*Taxol is more potent in YPD plates than in liquid media* — We speculate that substances present in peptones help to solubilize Taxol. Notably, Taxol is completely ineffective in SC media plates, even with the addition of potential solubilizing compounds like methyl cellulose or cyclodextrin, or after neutralizing pH with sodium succinate (data not shown).

*Filter sterilize YPD —* A large source of variability in the Taxol sensitivity spotting assay, and in liquid culture assays using YPD media, results from autoclaving YPD-based media. Differences in the heating temperature achieved, duration, and cooling rates alters the subsequent effectiveness of Taxol. In general, higher temperatures and/or delayed cooling reduces the effectiveness of Taxol added to the media. To avoid this, we vacuum filter-sterilize (.22 μm) 2× liquid YPD and mix with 2× autoclaved agar while still hot, and then subsequently add the desired amount of Taxol.

*Use a consistent peptone brand and lot number* — Taxol potency is sensitive to the source of peptone used in YPD media and plates. Indeed, with some peptones, Taxol can be ineffective. To ensure consistency, we recommend sticking with a validated peptone source and purchasing enough of a single lot to provide stability over the required time frame. After testing over 10 sources, we have had the best results with BD-brand Bacto™ peptone.

*Drug-sensitized yeast are sensitive to the source of peptone in YPD media* — Even in the absence of Taxol, the Taxol-sensitized yeast strains (and likely all drug-sensitized strains) are sensitive to the source of peptone used for YPD media. Indeed, we have found that drug-sensitized yeast will not grow on YPD made with some peptones even though wildtype yeast grows normally. Presumably this is because they are unable to export toxic elements in the media that wild type cells can tolerate or export. Thus, we recommend sticking with a validated peptone source and purchasing enough of a single lot to provide stability over the required time frame.

*Plate age affects Taxol potency —* The age of Taxol-containing YPD plates is another source of variation in sensitivity assays. Taxol plates that are more than several days old (e.g., 1–6 weeks) display increased potency compared to freshly made plates (data not shown). At present we attribute this phenomenon to the saturation or equilibration of Taxol-solubilizing factors throughout the plate, or breakdown products in the YPD contributing to Taxol solubilization. Regardless, all Taxol sensitivity spotting assays should be conducted with plates prepared and spotted in the same day for consistency.

### Live cell imaging using Taxol

For live-cell imaging of Taxol-sensitive strains (e.g., for MT dynamics or chromatid oscillation) we use a simple wet mount with no agarose pad. It is critical to realize that standard use of an agarose or agar pad (or material with similar properties) will significantly increase the effective potency of Taxol relative to in liquid cultures. This is similar to the increased potency seen on YPD + agar plates relative to liquid media. As an example, when MT dynamics were measured in cells on a 1.2% agarose pad in the presence of 30 μM Taxol, the overall dynamicity is only 2% of that seen in cells without Taxol, as opposed to 25% using 30 μM Taxol in the absence of an agarose pad (Table 3).

**Table 3:** Parameters of microtubule dynamic instability for cytoplasmic microtubules *in vivo*

	Control (no Taxol)^a^	Taxol (no pad)^a^	Taxol (1.2% agarose pad)^b^
Polymerization Rate (μm/min)	1.42 ± 0.62 (36)	0.72 ± 0.35 (13)∗∗∗∗	0.83 ± 0.69 (2)
Depolymerization Rate (μm/min)	2.10 ± 0.79 (33)	0.65 ± 0.23 (13)∗∗∗∗	1.27 ± 0.13 (2)
Catastrophe Frequency (min^−1^)	0.81 (28)	0.23 (9)	0.03 (2)
Rescue Frequency (min^−1^)	0.65 (12)	0.71 (9)	1.3 (2)
Time spent polymerizing, %	45.8	26.2	2.1
Time spent depolymerizing, %	34.7	24.3	1.0
Time spent attenuated, %	19.5	49.5	96.9
Average polymerization duration, s	40.7 ± 18 (36)	63.1 ± 34 (13)∗	21.0 ± 4 (2)∗
Average depolymerization duration, s	33.6 ± 17 (33)	58.5 ± 24 (13)∗∗	45.0 ± 30 (2)
Average attenuation duration, s	29.6 ± 11 (21)	103.3 ± 87 (15)∗∗	105.1 ± 70 (39)∗
Dynamicity, tubulin/sec	38.9	9.8	0.9
Total microtubule observed	37	39	39
Total time observed, s	3196	3130	4230

Sample number shown in parenthesis; Error = SD; ∗p < 0.05, ∗∗p < 0.01, ∗∗∗∗p < 0.0001 versus control by unpaired, two-tailed t-test.

a Previously reported in Proudfoot et al.^1^

b Measured concurrently with rates reported in Proudfoot et al.^1^

## Troubleshooting

### Problem 1

The cells are not synchronously releasing from G1 arrest (G1 release assay, steps 4–6).

### Potential solution

Decrease the amount of alpha-factor used for the arrest or increase the number/volume of Milli-Q water rinses when washing out the alpha-factor. Also, ensure that the cells are in exponential growth prior to alpha-factor addition and that a tight arrest was achieved, i.e., >95% arrested.

### Problem 2

The cells are not synchronously releasing from metaphase arrest with Cdc20 depletion (Cdc20 release assay, steps 28–33).

### Potential solution

The effective potency of the methionine-inducible arrest depends on the effective concentration of methionine per cell, as yeast cells likely sequester excess methionine which affects subsequent methionine-washout dependent release. In essence, using excess methionine to suppress *CDC20* expression, or even using lower concentrations in larger volume and/or more dilute cultures, results in slower release from metaphase arrest following washout. Conversely, using less methionine per cell results in more rapid release. If the release is not synchronous and abrupt, but appears to drag out, try decreasing the amount of methionine solution used (e.g., 1:25 or 1:50 dilution instead of 1:10) to arrest the culture in metaphase. The volume of methionine and density of the metaphase arrest culture can affect the speed of release (Figure 2). If scaling up the volumes in this assay, remember the amount of methionine used in the metaphase arrest can affect the speed and synchronicity of metaphase release into anaphase. Keep the ratio of total methionine (i.e., concentration ∗ volume) to cell number like in the original Cdc20 release volumes and adjust accordingly if needed.

### Problem 3

Cells for the Cdc20 release assay are not growing in overnight cultures, arresting when they should not arrest, or taking a long time to grow up in the overnight culture (Cdc20 release assay, steps 20–21).

### Potential solution

Ensure the cells are being cultured in SC-MET media and not SC media containing methionine. If methionine is in the media, the cells will arrest in metaphase and appear large budded. If there are even trace amounts of methionine in the media (as we found in some commercial media), cells will arrest by sequestering this methionine, especially when the cell density is very low as in following inoculation. This can present as a non-growing or slow-growing culture, or aberrant cell morphology.

### Problem 4

Cells do not seem to be sensitive to Taxol (G1 and Cdc20 release assays).

### Potential solution

First, try using a fresh tube or stock of methyl cellulose for the cultures and ensure the methyl cellulose is being used at 0.02% in the culture, and is added to the media prior to Taxol addition. If you are using fresh methyl cellulose and the cells still do not seem sensitive, check the 1 mM Taxol stock solution, and ensure it is completely and solidly frozen at −20°C. If not, make fresh 1 mM Taxol stock.

## Resource availability

### Lead contact

Further information and requests for resources and reagents should be directed to and will be fulfilled by the lead contact, Mohan Gupta (mgupta@iastate.edu).

### Materials availability

Taxol and drug-sensitive yeast strains (see Table S1) used for these studies are available by request (mgupta@iastate.edu).

## Data Availability

The published article includes all G1 and Cdc20 release assays, the Taxol sensitivity spotting assay, and microtubule dynamics data generated or analyzed during this study.

## Supplemental information

Table S1. Drug and Taxol-sensitive strains used in Figures and Tables
