# Supplementary material for: Using Taxol-sensitized budding yeast to investigate the effect of microtubule stabilization on anaphase onset
Source: STAR Protoc. 2023 Aug 18;4(3):102522. doi: 10.1016/j.xpro.2023.102522 (PMC10469069; doi:10.1016/j.xpro.2023.102522)
Supplement: Table S1. Drug and Taxol-sensitive strains used in Figures and Tables [file mmc1.pdf]

Supplemental Table 1: Yeast strains used, related to G1 release assay Step 1, Cdc20 release assay Step 20, and Taxol sensitive spotting assay Step 54.

Strain	Genotype	Figure
MGY1293	MAT**a**, *tub2-25*(A19K-T23V-G26D-Y270F), *erg6::HIS3, pdr1::hygB, pdr3::NAT, his3Δ200, leu2Δ1, ura3-52, trp1Δ63, pMET3-3xHA-CDC20-TRP1*	2, 3
MGY2150	MAT**a**, *tub2-25*(A19K-T23V-G26D-Y270F), *erg6::TRP1, pdr1::hygB, pdr3::NAT, his3Δ200, leu2Δ1, ura3-52, trp1Δ63*	1, 3, Table 3
MGY1872	MAT**a**, *TUB2, erg6::TRP1, pdr1::hygB, pdr3::NAT, his3Δ200, leu2Δ1, ura3-52, trp1Δ63*	4
MGY1874	MAT**a**, *tub2-25*(A19K-T23V-G26D-Y270F), *erg6::TRP1, pdr1::hygB, pdr3::NAT, his3Δ200, leu2Δ1, ura3-52, trp1Δ63*	4
MGY2104	MAT**a**, *tub2-25*(A19K-T23V-G26D-Y270F), *erg6::TRP1, pdr1::hygB, pdr3::NAT, his3Δ200, leu2Δ1, ura3-52, trp1Δ63, bub3::G418*	4
